# The teaching dilemma of health education teachers in China and path exploration: a cross-sectional study of Guangdong Province

**DOI:** 10.3389/fpubh.2025.1607420

**Published:** 2025-07-02

**Authors:** Zhen Wang, Lin Zhu

**Affiliations:** ^1^School of Sport and Health, Guangzhou Sport University, Guangzhou, China; ^2^Research Center for Innovative Development of Sports and Healthcare Integration, Guangzhou Sport University, Guangzhou, China; ^3^Innovative Research Center for Sports Science in the Guangdong-Hong Kong-Macao Greater Bay Area, Guangzhou Sport University, Guangzhou, China

**Keywords:** school-based health education, health literacy, health education teachers, teaching difficulty, cross-sectional study

## Abstract

**Background:**

School-based health education is a major way to improve the health literacy of children and adolescents, the teaching difficulty of health education teachers will directly affect its implementation. This study aimed to understand the degree of teaching difficulty of health education teachers in Guangdong Province and to explore the influencing factors.

**Methods:**

A self-made questionnaire was used to survey health education teachers of 232 primary and secondary schools in Guangdong Province. The survey included three components: teachers’ basic information, teaching situation, and school level investigation. Data were analyzed using the chi-square test for single factors and a binary logistic regression model for multiple factors.

**Results:**

A total of 5,416 (97.69% were valid) teachers responded to the questionnaire in the survey, and 44.26% were health education teachers. Health education teachers who were engaged in full-time jobs and had health-related training experience, and health education teachers whose schools provided uniform teaching materials and put a high value on health education, were less difficult to teach. Health education teachers whose schools had teaching standards and evaluation criteria were more difficult to teach. Among these factors, the occupation type and health-related training experience were the main factors affecting teaching difficulty.

**Conclusion:**

The teaching difficulty of health education teachers in primary and secondary schools in Guangdong Province is high. Strategies for building full-time health education teacher groups and enhancing health education training for teachers should be adopted to reduce teaching difficulty.

## 1 Introduction

Health literacy is a critical component for the development of social civilization. It is defined as “the degree to which individuals can obtain, process and understand basic health information and services needed to make appropriate health decisions” (1). Higher health literacy and numeracy skills are associated with better clinical outcomes and lower medical burdens (2, 3). How to effectively improve the health literacy of citizens has always been a global issue (1, 4, 5). In the context of “Healthy China 2030,” the Chinese government formulated a series of policies to promote the improvement of citizens’ health literacy (6–8). For instance, the “Healthy China 2030″ plan proposes that health education should be incorporated into the education system and made an important part of quality-oriented education at all levels. However, some problems and obstacles occur in the process of implementation. Finding the existing problems and proposing possible solutions may help to achieve “Healthy China”.

Childhood and adolescence are regarded as crucial periods for healthy development since fundamental cognitive, physical and emotional development, and health-related behaviors and skills are gradually formed during these stages (9). Appropriate health literacy is essential for personal health and well-being throughout adulthood (10–12). However, at the present stage of China, children and adolescents have many health problems. For example, the myopia rate in China has shown an increasing trend, approximately 80% of students completing secondary schooling are myopic, and more than 10% of them are highly myopic (13–15). Moreover, the prevalence of overweight and obesity were gradually increased in China (16, 17). Lower health literacy is one of the factors contributing to the rise in these problems (18, 19). Therefore, health education, especially aimed at school-aged children and adolescents, is urgently needed in China.

School-based health education is the main way for children and adolescents to acquire health-related knowledge (20, 21). Health education teachers are the implement principal part of school-based health education, their teaching difficulty will directly affect teaching quality, and further influences the development of students’ health literacy. However, previous studies mostly focused on the status of students’ health literacy (22, 23), the teaching situation of health education teachers is lack of research. Considering the essential role of health education teachers in school-based health education, studies are needed to understand the teaching difficulty and its influencing factors, which may help to formulate corresponding strategies and promote the development of school-based health education and the improvement of students’ health literacy.

In this study, we performed a cross-sectional survey among health education teachers in 21 prefectural-level cities in Guangdong Province to understand the teaching difficulty of health education teachers and the influencing factors. The objective of this study was to provide a reference point for reducing teaching difficulty of health education teachers and improving the development of health education in Guangdong Province. The findings might help develop health education in primary and secondary schools in China and provide strong evidence for other scholars to study the similarities and differences in school-based health education between China and other countries.

## 2 Methods

### 2.1 Study design and setting

A cross-sectional survey using multistage stratified sampling method was performed between September 2022 and November 2022 in Guangdong, China. The questionnaire survey was conducted among primary and secondary school teachers in 21 prefectural-level cities of Guangdong Province: Guangzhou, Shenzhen, Zhuhai, Shantou, Foshan, Shaoguan, Heyuan, Meizhou, Huizhou, Shanwei, Dongguan, Zhongshan, Jiangmen, Yangjiang, Zhanjiang, Maoming, Zhaoqing, Qingyuan, Chaozhou, Jieyang, and Yunfu. Specifically, one municipal district and one county were selected in each prefecture-level city. Two elementary schools, two middle schools, and two high schools were randomly selected in each district/county (one in urban areas and one in townships). The specific survey schools were determined by the Education Bureau of each district/county. After determining the survey school, one class was randomly selected from each grade, and all teachers of the sampled class were taken as questionnaire respondents.

### 2.2 Participants

The following criteria were established for participant selection. The inclusion criteria were as follows: (1) engaged in frontline teaching work and (2) volunteered to engage in this research. Participants who were not currently engaged in teaching work were excluded from the study. Additionally, to identify the health education teacher, the following question was asked: “In the last academic year, did you undertake or participate in the teaching of health education?.” Teachers who answered “yes” were considered to be health education teachers. The sample size calculation was based on the events per variable (EPV) criterion, which requires at least 10 outcome events for each independent variable in logistic regression to ensure model stability. This study included 10 independent variables, with health education teachers comprising 44.26% of the sample. The theoretically required minimum sample size was approximately 226 participants. A total of 5,291 participants (including 2,342 health education teachers) were ultimately included, meeting the sample size requirements for regression modeling.

Overall, a total of 5,416 questionnaires were collected in this survey, of which 5,291 were valid, with an effective rate of 97.69%. Among them, 2,342 were health education teachers, for a rate of 44.26%. More of the participants were from cities (51.58%) or the Pearl River Delta (41.76%), and more than half of them were female (64.26%). Most of them had education level of college (94.58%) and engaged in a part-time job of health education (86.21%). The specific details can be viewed in Table 1.

**Table 1:** Descriptive statistics of the study sample.

Variable	Subgroups	*N*	%
Area	City	1,208	51.58%
	Rural	1,134	48.42%
Region	Pearl River Delta	978	41.76%
	East	485	20.71%
	West	340	14.52%
	North	539	23.01%
Gender	Male	837	35.74%
	Female	1,505	64.26%
Age (years)	≤25	138	5.89%
	25 < n ≤ 35	577	24.64%
	35 < n ≤ 45	984	42.02%
	>45	643	27.46%
Teaching age (years)	≤10	643	27.46%
	10 < n ≤ 20	708	30.23%
	20 < n ≤ 30	731	31.21%
	>30	260	11.10%
Grade	Primary school	863	36.85%
	Junior high school	800	34.16%
	High school	679	28.99%
Education	High school or below	10	0.43%
	College	2,215	94.58%
	Master or above	117	5.00%
Occupation type	Full-time	323	13.79%
	Part-time	2019	86.21%

### 2.3 Questionnaire

The questionnaire used in the survey (Supplementary File 1) was designed based on the research purpose. The aim of this study was to investigate the degree of teaching difficulty of health education teachers and to analyze the factors that influence it. To ensure the scientific and logical nature of the questionnaire, the entire questionnaire was discussed with team members.

The questionnaire comprised three sections: (1) basic information of teachers, such as gender, age, education, and occupation type, et al.; (2) teaching situation, such as teaching grade and the degree of teaching difficulty. (3) school level investigation, such as the degree of value for health education in schools, whether the school provides uniform teaching materials, and whether the school has formulated uniform teaching standards and evaluation criteria.

To minimize potential sources of bias, several measures were implemented. First, randomized sampling procedures were employed to ensure representative participant selection. Second, participants were informed of the scientific significance and societal value of the research to enhance response authenticity and data accuracy. Third, questionnaires were administered through anonymous online platform to mitigate social desirability bias. Finally, data from participants with incomplete or abnormal responses were systematically excluded before statistical analysis. These strategies were designed to strengthen the validity and reliability of the study while building a solid basis for subsequent analysis.

### 2.4 Variables

#### 2.4.1 Dependent variable

The principal outcome of interest was the degree of teaching difficulty, which was split into five levels: very easy, easy, average, difficult, or very difficult. Due to the small number of teachers in some categories, the first two categories (very easy and easy) were merged into the “easy” category, and the latter three categories (average, difficult, and very difficult) were merged into the “difficult” category to avoid the impact of uneven data distribution on the results. Therefore, two categories were used in the analysis.

#### 2.4.2 Independent variables

Independent variables included area, region, education level, occupation details (teaching grade, occupation type, health-related training experience), and school situation (uniform teaching materials, teaching standards, teaching evaluation criteria, degree of value for health education in schools). Among these variables, regional classification was determined by geographic location within Guangdong Province. Specifically, Dongguan, Foshan, Guangzhou, Huizhou, Jiangmen, Shenzhen, Zhaoqing, Zhongshan, and Zhuhai were categorized as the Pearl River Delta region; Chaozhou, Jieyang, Shantou, and Shanwei were categorized as the East region; Maoming, Yangjiang, and Zhanjiang were categorized as the West region; Heyuan, Meizhou, Qingyuan, Shaoguan, and Yunfu were categorized as the North region. Additionally, the education level in the questionnaire originally comprised six categories: high school or below, technical school education, college, university, master, and doctor. To avoid the potential issue of uneven data distribution and its impact on the results, we consolidated “high school or below” and “technical school education” into a single category of “high school or below” based on the distinctive features of China’s education classification system. Similarly, “college” and “university” were merged into the “college” category, and “master” and “doctor” were combined into the “master or above” category. Finally, the degree of value for health education in schools was assessed with the following question: “How much importance does your school place on health education?” For analytical purposes, the first two categories (very important and important) were merged into the “important” category, the average category remained unchanged, and the latter two categories (not too important and not important) were merged into the “not important” category.

### 2.5 Statistical analysis

The data were analyzed using SPSS version 26.0 (SPSS, Chicago, Illinois, United States). We present descriptive statistics. The results of categorical variables were presented as the numbers and proportions, the chi-square test was used to study the differences in teaching difficulty. The binary logistic regression analysis was used to investigate factors affecting the teaching difficulty among health education teachers. *p* value < 0.05 was considered statistically significant.

## 3 Results

### 3.1 Teaching difficulty of teachers with different characteristics

The results of the chi-square test are presented in Table 2. Health education teachers with different characteristics have different responses to teaching difficulties. Specifically, the differences in teaching difficulty among health education teachers based on the 6 independent variables were statistically significant (*p* < 0.05), such as occupation type, health-related training experience, the degree of value for health education in schools, and whether school has uniform teaching materials, teaching standards, and evaluation criteria. Besides, the independent variables base on area, region, grade, and education level were not statistically significant (*p* > 0.05).

**Table 2:** Participants’ demographic characteristics.

Variable	Subgroups	Difficulty of teaching				X^2^	*p* value
		Hard (*n*, %)		Easy (*n*, %)			
Total		1879	80.23%	463	19.77%		
Area	City	968	80.13%	240	19.87%	0.015	0.902
	Rural	911	80.34%	223	19.66%		
Region	Pearl River Delta	783	80.06%	195	19.94%	0.139	0.987
	East	392	80.82%	93	19.18%		
	West	272	80.00%	68	20.00%		
	North	432	80.15%	107	19.85%		
Grade	Primary school	690	79.95%	173	20.05%	2.906	0.234
	Junior high school	656	82.00%	144	18.00%		
	High school	533	78.50%	146	21.50%		
Education level	High school or below	10	100.00%	0	0.00%	2.757	0.252
	College	1773	80.05%	442	19.95%		
	Master or above	96	82.05%	21	17.95%		
Occupation type	Full-time	240	74.30%	83	25.70%	8.299	0.004
	Part-time	1,639	81.18%	380	18.82%		
Health-related training experience	Yes	1,596	78.85%	428	21.15%	17.815	0.000
	No	283	88.99%	35	11.01%		
Uniform teaching materials	Yes	983	78.26%	273	21.74%	6.602	0.010
	No	896	82.50%	190	17.50%		
Teaching standards	Yes	884	77.82%	252	22.18%	8.103	0.004
	No	995	82.50%	211	17.50%		
Teaching evaluation criteria	Yes	958	77.76%	274	22.24%	10.005	0.002
	No	921	82.97%	189	17.03%		
Degree of value for health education in schools	Important	1,599	78.50%	438	21.50%	30.234	0.000
	Normal	263	92.28%	22	7.72%		
	Not important	17	85.00%	3	15.00%		

### 3.2 Analysis of the factors influencing the teaching difficulty of health education teachers

A binary logistic regression analysis was performed to analyze the factors influencing the teaching difficulty of health education teachers in Guangdong Province. As shown in Table 3, the results revealed that occupation type and health-related training experience were the factors affecting the teaching difficulty. Specifically, health education teachers who engaged in full-time job of health education and had health-related training experience were rated their teaching difficulty as low.

**Table 3:** Analysis of the factors influencing the teaching difficulty of health education teachers.

Variable	Subgroups	β	SE	Wald x^2^	*p* values	Odds ratio	95% CI
Occupation type	Full-time	-	-	-	-	-	-
	Part-time	−0.309	0.142	4.704	0.030	0.735	0.556–0.971
Health-related training experience	No	-	-	-	-	-	-
	Yes	−0.626	0.190	10.867	0.001	0.535	0.369–0.776
Uniform teaching materials	No	-	-	-	-	-	-
	Yes	−0.125	0.111	1.265	0.261	0.882	0.709–1.098
Teaching standards	No	-	-	-	-	-	-
	Yes	−0.071	0.141	0.257	0.612	0.931	0.707–1.227
Teaching evaluation criteria	No	-	-	-	-	-	-
	Yes	−0.120	0.145	0.678	0.410	0.887	0.667–1.180
Degree of value for health education in schools	Important	-	-	-	-	-	-
	Normal	0.683	0.669	1.044	0.307	1.980	0.534–7.341
	Not important	−0.371	0.634	0.343	0.558	0.690	0.199–2.389

## 4 Discussion

Childhood and adolescence are critical periods for establishing health concepts and healthy behaviors, the healthy habits formed during these periods will have a profound impact on one’s life (10–12). School-based health education is an important way to improve the health literacy of children and adolescents (20, 21), the teaching difficulty of health education teachers will directly affect the successful implementation of school-based health education. Therefore, understanding the teaching difficulty of health education teachers and the influencing factors may help to formulate corresponding countermeasures, and further promote the improvement of health education in schools. In this study, we take Guangdong Province as an example and performed a survey in primary and secondary schools, the results revealed that 80.23% of health education teachers had difficulties in teaching, and occupation type and health-related training experience were the influencing factors.

Teachers are among the most important factors in promoting students’ learning and academic achievement (24), their teaching difficulty will affect teaching effects. With a test of chi-square, we found that health education teachers with different characteristics were having significant differences in teaching difficulty. Specifically, health education teachers who were engaged in full-time jobs and had health-related training experience, and health education teachers whose schools provided uniform teaching materials and put a high value on health education, were less difficult to teach. The results indicated that the use of uniform teaching materials for lesson preparation may help to reduce the difficulty of teaching. Health education can be divided into two categories: formal health education and informal health education (25). Formal health education refers to a health education lesson led by a well-trained health education teacher. Informal health education includes bulletin boards, newsletters, and school health office webpages. Among them, formal health education is more important for the development of children’s and adolescents’ health literacy. Therefore, using uniform teaching materials to prepare lessons and make lesson plans is helpful for improving the quality of formal health education, which is urgently needed at present stage. However, only 53.58% of primary and secondary schools in Guangdong Province have uniform teaching materials. Education authorities should take the lead in organizing a team of experts at the Guangdong provincial level to compile unified health education teaching materials for children and adolescents at different stages. Besides, we also surprisingly found that health education teachers whose schools have teaching standards and evaluation criteria were more difficult to teach, which suggested that the setting of teaching standards and evaluation criteria can enable teachers to conduct self-inspects, and the improvement of their own teaching requirements may make them feel difficult to teach. Additionally, it should be noted that no significant differences in teaching difficulty were observed among health education teachers across different areas and regions. However, study have shown that students from urban cities in China have higher health literacy than those from rural areas (26). Moreover, study in other countries have also found that populations in economically developed regions have higher health literacy levels than those in less developed areas (27). This discrepancy may be due to the objective nature of health literacy assessment, while the evaluation of teaching difficulties among health education teachers is based on subjective self-ratings. Although we informed participants of the scientific significance and societal value of the research and conducted the survey using an online anonymous questionnaire, potential subjectivity-induced bias among health education teachers may still exist. Future studies should incorporate objective assessments of actual teaching outcomes to enable more objective and accurate analysis. Nevertheless, our findings still revealed the possible causes of teaching difficulty faced by health education teachers. Educational institutions and administrative authorities should implement targeted interventions to address the teaching difficulty.

Furthermore, to understand the influencing factors of teaching difficulty, we performed a binary logistic regression analysis and found that occupation type and health-related training experience were the factors affecting the teaching difficulty. However, at present, only 13.79% of health education teachers can work full-time, and more than 80% of them are required to take on other duties. In fact, the health education in school is mostly undertaken by physical education teachers in China. Therefore, enhancing the health education competency of physical education teachers and promoting their professional transition toward becoming physical education and health teachers is of great importance. Liu et al. (28) developed a model to assess the health service competence of Chinese physical education teachers across three dimensions: health service beliefs, foundational health knowledge, and health service skills. Education authorities could adopt this framework to conduct periodic competency evaluations for physical education teachers, in order to improve their health literacy and teaching ability. More importantly, the education authorities should strive to build a full-time health education teacher group in primary and secondary schools and formulate corresponding policies to guarantee full-time work. In addition, although 80.23% of health education teachers have had health-related training experience, the vast majority of teachers still feel difficult to teach. As has been recognized by many research, health education training can enhance teachers’ knowledge, attitudes, health self-efficacy, and teaching skills (29–31), and it is associated with students’ health behaviors (32, 33). Notably, while social media facilitates public access to health information, a lack of basic health knowledge and discrimination may lead to misguided health behaviors when exposed to online health misinformation (34). Therefore, the education authorities should regularly organize diverse training activities, such as lectures, training camps, and online courses, to ensure that health education teachers can access accurate health information.

This study has several limitations that should be noted. First, even though participants were informed of the research’s scientific significance and societal importance, and anonymous online surveys were employed, the assessment of teaching difficulty remained inherently subjective. This reliance on self-reported evaluations may have introduced measurement bias due to the absence of objective validation metrics. Second, although municipal districts and counties were randomly selected from each prefecture-level city, survey schools were selected by the local Education Bureau to ensure full cooperation. This non-random school selection may have compromised sampling randomness and potentially introduced selection bias. Third, the teaching difficulty were consolidated into two categories (low and high) due to insufficient sample sizes in certain categories. This categorization approach may have influenced analytical outcomes. Future studies should expand sampling range to include more schools and teachers, thereby enhancing the statistical robustness of findings.

## 5 Conclusion

Reducing teaching difficulty of health education teachers is one of the important ways to promote the implementation of school-based health education and improve health literacy of school-aged children and adolescents. The teaching difficulty of health education teachers in primary and secondary schools in Guangdong Province is high, and occupation type and health-related training experience are the influencing factors. Efforts should be made at the education department level to reduce the teaching difficulty of teachers. Education authorities should build a full-time health education teacher group in primary and secondary schools, and simultaneously enhance the health education training for teachers.

## Data Availability

The raw data supporting the conclusions of this article will be made available by the authors, without undue reservation.
