# Supplementary material for: The teaching dilemma of health education teachers in China and path exploration: a cross-sectional study of Guangdong Province
Source: Front Public Health. 2025 Jul 2;13:1607420. doi: 10.3389/fpubh.2025.1607420 (PMC12263607; doi:10.3389/fpubh.2025.1607420)
Supplement: Supplementary file 1 [file Table_1.docx]

Supplementary Material

**Questionnaire on the Teaching of Health Education in Primary and Secondary Schools**

Dear Teacher:

Greetings! You are invited to participate in the "Survey on the Implementation Status of Health Education in Primary and Secondary Schools in Guangdong Province". This survey is conducted by the Guangdong Provincial Department of Education and organized by Guangzhou Sport University. The aim is to understand the implementation status of health education in primary and secondary school, so as to promote and strengthen the work of health education in Guangdong Province. Please fill in the answers according to your actual situation. Your objective and truthful answers will help our province to guide the work of health education in primary and secondary schools. Thank you for your cooperation.

Before you fill in this questionnaire, please pay attention to the following matters:

1. Each question in the questionnaire is important, please answer each question, do not miss.

2. There are no right or wrong answers, please fill in the questionnaire according to the actual situation.

3. We promise that all information will be kept strictly confidential and used only for policy and academic research.

**I. Basic information**

1. Your school: _________ city _________ county (district) ____________ school name [Fill in the blank] *

2. Your gender: [Single choice] *

| ○Male | ○Female |  |  |  |  |  |  |
| --- | --- | --- | --- | --- | --- | --- | --- |

3. Your date of birth: [Fill in the blank] *

_________________________________

4. Your teaching experience (years): [Fill in the blank] *

Fill in a whole number of years of teaching experience, e.g., 10

_________________________________

5. In the last academic year, did you undertake or participate in the teaching of health education? [Single choice] *

| ○Yes |
| --- |
| ○No (Please skip to Question 8) |

6. Are you a full-time or part-time health education teacher? [Single choice] *

| ○Full-time (Please skip to Question 7) |
| --- |
| ○Part-time (Please skip to Question 8) |

Dependent on the 1st option of question 5

7. Do you have any other teaching responsibilities outside of health education teaching? [Single choice] *

| ○Yes (Please skip to Question 8) |
| --- |
| ○No |

8. Which subject do you primarily teach? [Single choice] *

| ○Chinese |
| --- |
| ○Math |
| ○English |
| ○Foreign Languages (small languages other than English) |
| ○Health Education |
| ○History |
| ○Geography |
| ○Physics |
| ○Chemistry |
| ○Biology |
| ○Quality Education |
| ○Ideological and Moral (or: Moral Character and Life, Moral Character and Society) |
| ○Physical Education |
| ○Arts |
| ○Music |
| ○Computer (or: Information Technology) |
| ○Artificial Intelligence |
| ○General Technology |
| ○Common sense of nature (or: Science) |
| ○Integrated Practical Activities |
| ○Labor Course |
| ○Other _________________ |

9. You have been teaching health education since _________? [Fill in the blank] *

Fill in an integer year, e.g., 2020

_________________________________

Dependent on the 1st option of question 5

10. Do you perform any of the following roles? [Multiple choice] *

| □Class teacher |
| --- |
| □Teaching and research team leader |
| □Group leader of lesson preparation |
| □Grade dean |
| □School Leader |
| □Others_________________ |

11. What is your politics status? [Single choice] *

| ○Members of the Communist Party |
| --- |
| ○Member of the Communist Youth League |
| ○Members of Democratic Party or Federation of Industry and Commerce |
| ○Nonpartisan |
| ○The masses |
| ○Other_________________ |

12. What is your job title? [Single choice] *

| ○No title |
| --- |
| ○Professor |
| ○First grade |
| ○Second grade |
| ○Third grade |
| ○Other _________________ |

13. What is your highest education? [Single choice] *

| ○High school or below (Please skip to Question 17) |
| --- |
| ○Technical School education (Please skip to Question 17) |
| ○College (Please skip to question 14) |
| ○University (Please skip to Question 14) |
| ○Master (Please skip to question 14) |
| ○Doctor (Please skip to question 14) |

14. Are you a normal school student? [Single choice] *

| ○Yes |
| --- |
| ○No |

15. What was your major in university (or college)? [Single choice] *

| ○Medicine or Health Sciences |
| --- |
| ○Social Sciences (Political, Psychological, Social, Journalism and Communication, etc.) |
| ○Business |
| ○Natural Sciences (Science, Engineering, Agriculture, etc.) |
| ○Humanities (literature, history, philosophy, etc.) |
| ○Art (music, dance, art, broadcasting, etc.) |
| ○Physical education |
| ○Legal |
| ○Other _________________ |

16. What was your major in graduate school? [Single choice] *

| ○Medicine or Health Sciences |
| --- |
| ○Social Sciences (Political, Psychological, Social, Journalism and Communication, etc.) |
| ○Business |
| ○Natural Sciences (Science, Engineering, Agriculture, etc.) |
| ○Humanities (literature, history, philosophy, etc.) |
| ○Art (music, dance, art, broadcasting, etc.) |
| ○Physical education |
| ○Legal |
| ○Other _________________ |

Dependent on the 5th;6th option of question 13

17. Have you taken any health education courses or participated in relevant training? [Single choice] *

| ○Yes |
| --- |
| ○No |

**II.** **Research on teaching situation**

18. What is the grade of the sample class in which you are teaching? [Single choice] *

| ○First grade of primary school |
| --- |
| ○Second grade of primary school |
| ○Third grade of primary school |
| ○Fourth grade of primary school |
| ○Fifth grade of primary school |
| ○Sixth grade of primary school |
| ○First grade of junior high school |
| ○Second grade junior high school |
| ○Third grade junior high school |
| ○First grade of high school |
| ○Second grade of high school |
| ○Third grade of high school |
| ○Other |

Dependent on the 1st option of question 5

19. What is the sample class in which you are teaching? [Fill in the blank] *

Fill in the integer class number, e.g., class 5 please fill in: 5

_________________________________

20. On average, how many hours per semester do you teach health education? [Fill in the blank] *

E.g., 20

_________________________________

Dependent on the 1st option of question 5

21. Do you think you have taken on too many health education teaching tasks? [Single choice] *

| ○Easy |
| --- |
| ○Relatively easy |
| ○Average |
| ○More |
| ○Too much |

Dependent on the 1st option of question 5

22. Do you have a lesson plan for your health education teaching? [Single choice] *

| ○Yes |
| --- |
| ○No |

Dependent on the 1st option of question 5

23. What do you think of the importance of school health education teaching to improve students' health literacy? [Single choice] *

| ○Not important |
| --- |
| ○Not too important |
| ○Average |
| ○Important |
| ○Very important |

24. Do you find it difficult to teach health education? [Single choice] *

| ○Very easy |
| --- |
| ○Easy |
| ○Average |
| ○Difficult |
| ○Very difficult |

Dependent on the 1st option of question 5

**III. School-level investigation**

25. How much importance does your school place on health education? [Single choice] *

| ○Very important |
| --- |
| ○Important |
| ○Average |
| ○Not too important |
| ○Not important |

26. Does your school use uniform teaching materials for health education teaching? [Single choice] *

| ○Yes (Please skip to Question 27) |
| --- |
| ○No (Please skip to Question 29) |

Dependent on the 1st option of question 5

27. Please write the name of the textbook used. [Fill in the blanks] *

_________________________________

Dependent on the 1st option of question 5

28. What do you think of the quality of the material? [Single choice] *

| ○Very bad |
| --- |
| ○Bad |
| ○Average |
| ○Good |
| ○Very good |

Dependent on the 1st option of question **5**

29. Are there uniform teaching standards for health education in schools? [Single choice] *

| ○No |
| --- |
| ○Yes |

Dependent on the 1st option of question 5

30. Are there uniform teaching evaluation criteria for health education in schools? [Single choice] *

| ○No |
| --- |
| ○Yes |

Dependent on the 1st option of question 5

31. What do you think is the biggest problem in carrying out health education at this stage? (If no, please fill in "none") [Fill in the blanks] *

_________________________________

32. Do you have any comments or suggestions for school on the teaching of health education? (If no, please fill in "none") [Fill in the blanks] *

_________________________________

**Ⅳ. Additional questions**

33. How do I address you (name or title)? [Fill in the blank] *

_________________________________

34. What is your telephone number? [Fill in the blank] *

_________________________________

35. What is your WeChat number? [Fill in the blank]

_________________________________

**Thank you for your support, wish you a happy life!**
